# Human Adipose Tissue-Derived Mesenchymal Stem Cells Target Brain Tumor-Initiating Cells

**DOI:** 10.1371/journal.pone.0129292

**Published:** 2015-06-15

**Authors:** Seung Ah Choi, Ji Yeoun Lee, Sung Eun Kwon, Kyu-Chang Wang, Ji Hoon Phi, Jung Won Choi, Xiong Jin, Ja Yun Lim, Hyunggee Kim, Seung-Ki Kim

**Affiliations:** 1 Division of Pediatric Neurosurgery, Pediatric Clinical Neuroscience Center, Seoul National University Children’s Hospital, College of Medicine, Seoul, Korea; 2 Adolescent Cancer Center, Seoul National University Cancer Hospital, Seoul, Korea; 3 Department of Anatomy, Seoul National University College of Medicine, Seoul, Korea; 4 Department of Bioengineering, School of Engineering and Applied Science, University of Pennsylvania, Philadelphia, PA, United States of America; 5 Cell Growth Regulation Laboratory, School of Life Sciences and Biotechnology, Korea University, Seoul, Korea; University of Florida, UNITED STATES

## Abstract

In neuro-oncology, the biology of neural stem cells (NSCs) has been pursued in two ways: as tumor-initiating cells (TICs) and as a potential cell-based vehicle for gene therapy. NSCs as well as mesenchymal stem cells (MSCs) have been reported to possess tumor tropism capacities. However, there is little data on the migratory capacity of MSCs toward brain tumor-initiating cells (BTICs). This study focuses on the ability of human adipose tissue derived MSCs (hAT-MSCs) to target BTICs and their crosstalk in the microenvironment. BTICs were isolated from three different types of brain tumors. The migration capacities of hAT-MSCs toward BTICs were examined using an *in vitro* migration assay and *in vivo* bioluminescence imaging analysis. To investigate the crosstalk between hAT-MSCs and BTICs, we analyzed the mRNA expression patterns of cyto-chemokine receptors by RT-qPCR and the protein level of their ligands in co-cultured medium. The candidate cyto-chemokine receptors were selectively inhibited using siRNAs. Both *in vitro* and *in vivo* experiments showed that hAT-MSCs possess migratory abilities to target BTICs isolated from medulloblastoma, atypical teratoid/rhabdoid tumors (AT/RT) and glioblastoma. Different types of cyto-chemokines are involved in the crosstalk between hAT-MSCs and BTICs (medulloblastoma and AT/RT: CXCR4/SDF-1, CCR5/RANTES, IL6R/IL-6 and IL8R/IL8; glioblastoma: CXCR4/SDF-1, IL6R/IL-6, IL8R/IL-8 and IGF1R/IGF-1). Our findings demonstrated the migratory ability of hAT-MSCs for BTICs, implying the potential use of MSCs as a delivery vehicle for gene therapy. This study also confirmed the expression of hAT-MSCs cytokine receptors and the BTIC ligands that play roles in their crosstalk.

## Introduction

Cancer stem cells (CSCs) or tumor-initiating cells (TICs) are defined as cancer cells that can self-renew and give rise to all other types of cancer cells [1]. These cells are thought to be responsible for tumor-initiation, propagation and chemo/radiation therapy resistance, making cancers relapse and difficult to cure [2]. In brain tumors, putative brain tumor-initiating cells (BTICs) from glioblastoma, medulloblastoma and ependymoma have been identified [3, 4]. These BTICs possess stem cell-like characteristics, including the expression of neural stem cell (NSC) markers such as nestin, musashi, SOX2, OLIG2, ZFX, and CD133 [5,6], capability of self-renewal, formation of neurosphere-like spheroids and differentiation into various nervous system lineages, such as neurons, astrocytes and oligodendrocytes. Furthermore, these cells are tumorigenic in *in vivo* serial transplantation and are able to generate xenograft tumors with the same biological and genomic features of the parental brain tumors [7]. Hence, targeting BTICs has been proposed as a novel cancer treatment that would allow for better prognoses of brain tumors [1].

In tumor targeting, mesenchymal stem cells (MSCs) with multi-lineage potential show a broad migratory capacity for brain tumors, including glioblastoma, medulloblastoma, ependymoma and astrocytoma [8–10]. Hence, they have been studied as a better alternative to NSCs, which have limited availability and ethical issues despite their strong tumor-tropic properties. Among the different types of MSCs, human adipose tissue-derived MSCs (hAT-MSCs) arise as one of the most attractive vehicles for the delivery of therapeutic agents in clinical applications because they are available in large amounts, easy to isolate and expand, free of ethical concerns and, most importantly, eligible for autologous transplantation [9, 10].

Although MSCs have been shown to target certain types of brain tumors [11, 12], not much study has been performed on their ability to migrate toward BTICs. Furthermore, the true migratory mechanism has yet to be clarified, and the crosstalk between hAT-MSCs and BTICs in a tumor microenvironment is not fully understood.

In the present study, we focused on the ability of hAT-MSCs to target BTICs and their crosstalk in the microenvironment. The migration capacity of hAT-MSCs for BTICs was evaluated using both *in vitro* and *in vivo* settings. Furthermore, the mRNA expression patterns of cyto-chemokine receptors and protein levels of their ligands in the microenvironment were analyzed using hAT-MSCs and BTICs co-culture systems.

## Materials and Methods

### Ethics and statement

Fresh brain tumor specimens and adipose tissue samples were collected from patients upon precedent written informed consent from themselves or their parents, which was reviewed and approved by the Institutional Review Board (IRB) of the Seoul National University Hospital (SNUCM/SNUH IRB 0606-049-176).

Animal experiments were approved by the Institutional Animal Care and Use Committee Review Boards of the Soul National University Hospital (IACUC 10–0262) and conducted in accordance with the National Institutes of Health Guide for the Care and Use of Laboratory Animals (NIH publication 80–23). All efforts were taken to minimize the suffering and stress of animals.

### Human samples

Tumor samples were prospectively obtained from pediatric medulloblastoma (N = 3), atypical teratoid/rhabdoid tumors (AT/RT, N = 2) and glioblastoma (N = 3) at the initial operation before any adjuvant therapy was performed (S1 Table). Adipose tissue samples were obtained from the abdominal fat prepared for sellar floor reconstruction in patients who had undergone transsphenoidal surgery.

### Cell cultures

Tumor tissues were used within 2 hours for primary cell cultures. After resection, tissues were washed and enzymatically dissociated. When the cells formed tumor spheres, they were plated without dissociation into extracellular matrix (ECM)-coated flasks (ECM 1:10 dilution, Sigma). The spheres were maintained, and subcultures were performed as described previously [7, 13]. Cells were isolated and cultured in NBE media consisting of neurobasal medium (Invitrogen, Grand Island, NY), glutamax (Invitrogen), N2 and B27 supplements (0.5× each; Invitrogen), human recombinant epidermal growth factor (EGF) and basic fibroblast growth factor (bFGF; both from Chemicon, Temecula, CA).

hAT-MSCs were cultured and expanded in minimum essential medium alpha (MEM α, Invitrogen) supplemented with 10% fetal bovine serum (FBS) and 1% antibiotic–antimycotic solution (Invitrogen) as described previously [10] All primary cultured cells were maintained and used before the fourth cell passage. The human fibroblast cell line HFF1 was purchased from American Type Culture Collection. All cells were incubated at 37°C in an incubator in a 5% CO_2_/95% air atmosphere.

### Characterization of primary cultured BTICs

To characterize the primary cultured BTICs, tumor spheres were induced and fixed onto poly-L-lysine–coated 8-well plates (Nunc, Rochester, NY) in NBE for 2–7 days. The numbers of tumor spheres were observed under an inverted microscope (Leica microsystem, Wetzlar, Germany). Tumor spheres were stained with primary antibodies directed against nestin (as a neuroectodermal stem-cell marker, 1:200; Chemicon), musashi (as a neural stem cell marker, 1:500; Millipore, Temecula, CA) and Sox2 (as a neural stem cell marker 1:200; Abcam, Cambridge, MA). The secondary antibodies Alexa Fluor 488-conjugated goat anti-mouse IgG and 594-conjugated goat anti-rabbit (1:200; Invitrogen) were used. Cells were mounted with an antifading solution containing 4′-6-diamidino-2-phenylindole (DAPI; Vector Laboratories, Burlingame, CA) for immunofluorescent staining. Fluorescent images were obtained using a confocal microscope (Zeiss, Oberkochen, Germany).

Dissociated cells (1×10^5^) from tumor spheres [medulloblastoma (N = 3), AT/RT (N = 2) and glioblastoma (N = 3)] were implanted into 8-week-old BALB/c nude mice using a stereotaxic device and a minimally traumatic technique (coordinates relative to the bregma: 1 mm anterior, 2 mm lateral, 3 mm depth) after anesthesia with a mixture of 10 mg/kg xylazine (Bayer Korea, Seoul, Korea) and 30 mg/kg Zoletil (Virbac, Carros, France) by intraperitoneal injection. Thirty days after cell implantation, the mice were perfused with 4% paraformaldehyde under deep anesthesia. Whole brains were fixed and dehydrated in graded sucrose concentrations. The tissues were embedded in OCT compound (Tissue-Tek, Torrance, CA) and stored at –80°C. The brains were sectioned coronally into 10-μm-thick slices using a cryostat and stained with hematoxylin and eosin.

### Characterization of primary cultured hAT-MSCs

We characterized the primary cultured cells from human adipose tissues. The cells were observed their morphology by inverted microscope and characterized using surface markers and fluorescence-activated cell sorting (FACS) analyses, with labeling by fluorescein-isothiocyanate (FITC)-conjugated monoclonal antibodies directed against the cluster of differentiation (CD) markers (BD Biosciences Pharmingen, San Diego, CA) CD73, CD90 or CD105 or with phycoerythrin (PE)-conjugated monoclonal antibodies directed against CD31, CD34 or CD45. Analysis was performed using a FACscan argon laser cytometer (Becton Dickinson, San Jose, CA). Additionally, the multipotency of the cells was determined using a mesenchymal differentiation kit (Invitrogen) as previously described [10]. The differentiation of hAT-MSCs into adipocytes, osteocytes and chondrocytes was confirmed by Oil Red O, Alizarin Red S and Alcian blue staining, respectively. All experiments were conducted in triplicate.

### In vitro migration assay

Dissociated tumor spheres (5×10^4^) from medulloblastoma (N = 3), AT/RT (N = 2), glioblastoma (N = 3) or HFF1 were added to the lower chamber of a Transwell support (8-μm pore size, Corning Costar, NY) in serum-free medium, and hAT-MSCs or HFF1 cells (5×10^4^) were plated into the upper chamber. After 6 hours, cells remaining at the upper surface were completely removed using a cotton swab. The migrated cells at the lower surfaces of the membranes were fixed and stained in a solution of 1% crystal violet in 2% ethanol for 30 seconds and afterwards rinsed with distilled water. The stained cells were soaked in ice-cold acetic acid, oscillated for 10 minutes and assessed by determining the absorbance at 570 nm using a micro-plate reader. The assays were performed in triplicate.

### Live imaging for in vivo tumor tropism

The migration of hAT-MSCs toward BTIC-derived tumors was confirmed and compared with fibroblasts using fluorescent magnetic nanoparticles (NEO-LIVE, Biterials, Korea) for *in vivo* live imaging. All live *in vivo* image acquisition and analysis were performed using an *in vivo* multispectral imaging system (CRi Maestro In-Vivo Imaging System).

At first, BTICs were labeled with NEO-LIVE 675 for 24 hours and suspended in PBS at a concentration of 1×10^5^ cells per 3 μl. Eight-week-old female nude mice (N = 6 for AT/RT-BTICs) were anesthetized as previously described. NEO-LIVE 675-labeled BTICs were implanted into the right forebrain of the mice (2.5 mm lateral and 1 mm anterior to bregma, at a 3 mm depth from the skull surface) by using a stereotaxic device and a minimally traumatic technique. One week after tumor cell injection, NEO-LIVE 797 labeled hAT-MSCs (2×10^5^ cells/3 μl) were stereotaxically injected into the left forebrain under the anesthesia. To detect the two color *in vivo* spectral fluorescence imaging, optical image sets were acquired with both a blue and deep red as described previously [14].

In the second step, the migration ability of hAT-MSCs was compared with that of HFF1 cells. Eight-week-old female nude mice (N = 3 for saline, N = 3 for medulloblastoma-BTICs, N = 6 for AT/RT-BTICs, N = 6 for glioblastoma-BTICs) were anesthetized as previously described. Unlabeled BTICs were implanted into the right forebrain of the mice (same coordinates) using a stereotaxic device and a minimally traumatic technique. After then, NEO-LIVE 797-labeled hAT-MSCs or HFF1 cells were injected into the left forebrain of the mice. The rainbow color-coded images of injected NEO-LIVE 797-labeled hAT-MSCs or HFF1 were processed (650 nm and 750 nm band-pass filters for excitation and emission, respectively) as described previously [15].

### Co-cultures

Dissociated tumor spheres (2×10^4^) from medulloblastoma (N = 3), AT/RT (N = 2) and glioblastoma (N = 3) were seeded in 2 ml of medium in 6-well plates, and 0.4-μm Transwell systems (Corning Costar) were placed into the wells. Then, hAT-MSCs or HFF1 cells (2×10^4^) were suspended in 2 ml of serum-free cell culture medium and added to each Transwell. Co-culture controls containing only culture media were incubated for 48 hours. Following incubation, the cell culture supernatants were collected, and the aliquots were stored at −80°C for the cytokine assay. For further studies, the cells were harvested to isolate RNA and protein. All experiments were conducted in triplicate.

### Real-time Quantitative Polymerase Chain Reaction (RT-qPCR)

After the hAT-MSCs were co-cultured with BTICs [medulloblastoma (N = 3), AT/RT (N = 2) and glioblastoma (N = 3)], total RNA was extracted from hAT-MSCs using the PureLink RNA mini kit (Invitrogen), and cDNA synthesis was performed using EcoDry Premix-oligo dT (Clontech, Mountain View, CA) following the manufacturer’s instructions. The mRNA levels of the selected genes were assessed by real-time quantitative PCR (RT-qPCR) using custom TaqMan array plates (Applied Biosystems, Carlsbad, CA) and an ABI7500 system. Probes were selected from inventoried gene expression assays (Invitrogen, S2 Table) and customized in triplicate. RT-qPCR analysis was performed using TaqMan probes for cyto-chemokine receptors and glyceraldehyde-3-phosphate dehydrogenase (GAPDH). The reactions were performed under conditions specified in the ABI TaqMan Gene Quantitation assay protocol and were repeated in triplicate. The comparative threshold cycle (Ct) method was used to calculate the relative gene expression. To quantify gene expression, the target genes were normalized to an endogenous reference (GAPDH). Relative quantification of target gene expression was calculated using the comparative Ct method. All experiments were conducted in triplicate.

### Cytokine array

After co-culture of hAT-MSCs and BTICs, cytokines were detected in the conditioned medium using the Bio-Plex cytokine assay system according to the manufacturer's protocol. Medium without cells was used as a negative control sample for cytokine analysis. Single hAT-MSCs or BTICs culture medium was obtained to analyze the basal cytokine levels. The concentrations of cyto-chemokines (S2 Table) were determined using Fluorokine MAP Base kits (R&D system, Minneapolis, MN), the hVersamap multiplex system (R&D system) and SDF-1 (CXCL4, R&D system) and IGF-1 (R&D system) ELISA kits according to the manufacturer's protocol. All experiments were conducted in triplicate.

The induced cytokine levels in the BTICs, HFF1 cell and hAT-MSCs co-cultured conditioned media were obtained as follows: [induced cytokine level in the BTICs with HFF1] = [total cytokine level in the BTICs with HFF1]-[cytokine level in the HFF1 only] and [induced cytokine level in the BTICs with hAT-MSCs] = [total cytokine level in the BTICs with hAT-MSCs]-[cytokine level in the hAT-MSCs only].

### Knock down of cytokine receptors on hAT-MSCs using siRNA

For siRNA transfection, hAT-MSCs were incubated the day before transfection in antibiotic-free media. CXCR4 (Cat. #1037826 and 1171128), CCR5 (Cat. #1172495 and 1172496), IGF1R (Cat. #100424 and 100425) and negative control siRNA (NC-siRNA, SN-1002) from Bioneer (Daejeon, Korea). The siRNAs (100 nM final concentration) were transfected using RNAi MAX (Invitrogen) following the manufacturer’s protocols. Experiments were performed 48 hours after transfection and the cells were allowed to recover for 24 hours prior to performing experiments. The knock down efficiency and specificity with siRNA was confirmed by western blotting. After knock down of cytokine receptors on hAT-MSCs, cell migration assay was performed as described above.

### Western blot analysis

BTICs cells co-cultured with hAT-MSCs were collected and lysed in protein lysis buffer (Cell Signaling, Danvers, MA). Equal amounts of sample lysate were separated by NuPAGE 4–12% bis-Tris gel (Invitrogen) and transferred to a nitrocellulose iblot gel transfer stack (Invitrogen) using the iblot system (Invitrogen). The membranes were blocked with 5% skim milk in Tris-buffered saline, Tween-20 (TBST) buffer and incubated overnight at 4°C with anti-SDF-1 (1:500, Abnova, Taipei, Taiwan), anti-RANTES (1:250, Abcam), anti-IL-6 (1:200, Thermo Scientific, IL), anti-IL-8, anti-IGF-1 (1:500, Abcam), anti-CXCR4R (1:1000, Abcam), anti-CCR5 (1:500, R&D system), anti-IGF1R (1:250, Abcam) and β-actin (1:5000, Sigma-Aldrich). The membranes were washed with TBST buffer and incubated with horseradish peroxidase-conjugated secondary antibodies. The membranes were developed with enhanced chemiluminescence kits (Invitrogen) and exposed to film. For quantification, the band densities were normalized to the levels of β-actin and represented as the relative intensity.

### Statistical analyses

All experiments were performed in triplicate, and the values were calculated as the means±standard deviation (SD) or expressed as a percentage of the controls±SD. Multiple group comparisons were performed by one-way ANOVA with a post hoc test. Differences between two groups were determined using a two-tailed Student *t*-test. The GraphPad Prism software (GraphPad Software, San Diego, CA) was used for all analyses. Differences were considered significant at p<0.05.

## Results

### Isolation and characterization of primary cultured BTICs and hAT-MSCs

**Fig 1:**
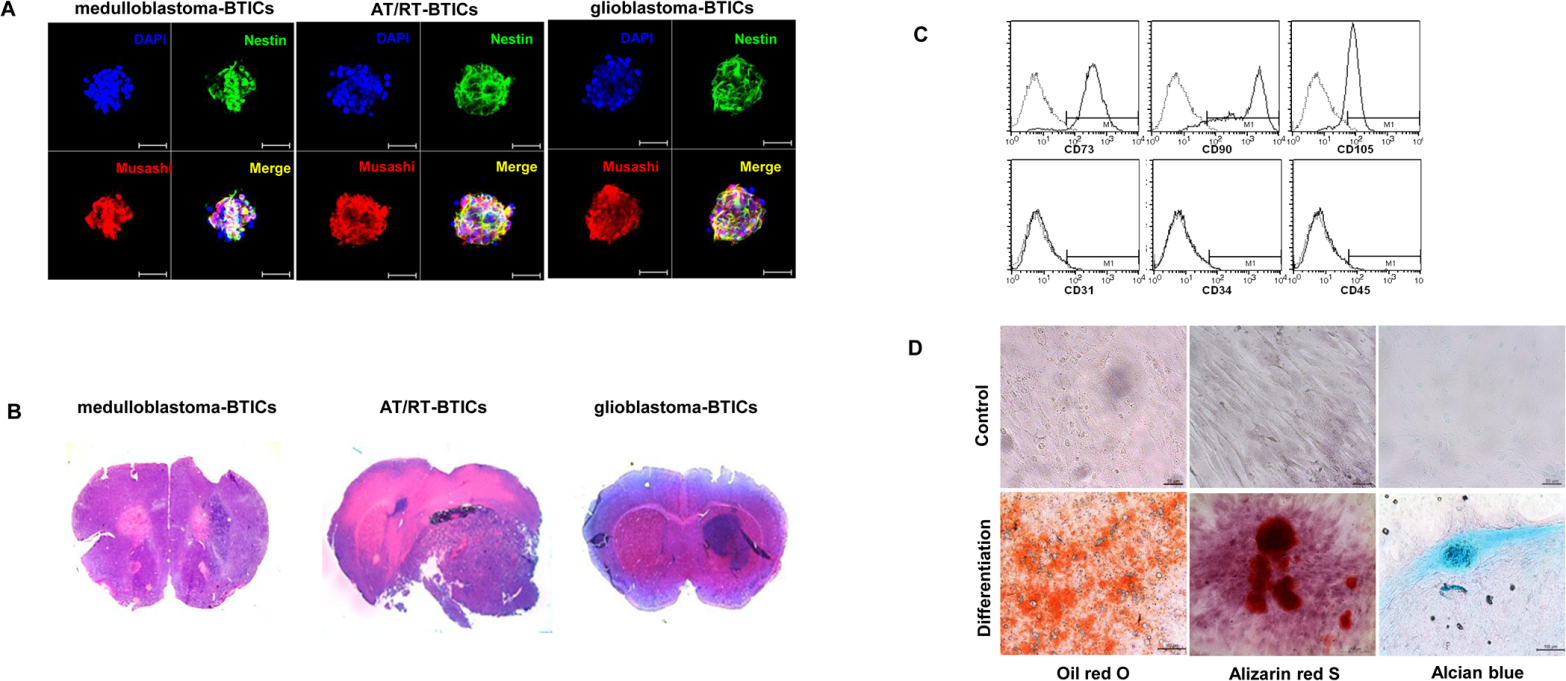
Characterization of brain tumor-initiating cells (BTICs) and human adipose tissue-derived mesenchymal stem cells (hAT-MSCs). (A) Tumor spheres show BTIC characteristics. Confocal microscopic analysis of tumor spheres stained with nestin and musashi. Scale bar, 50 μm. (B) The gross appearance of tumor formation in the nude mouse brain after injection of BTICs; ×1.25 magnification. (C) Flow cytometry analysis shows the immunophenotyping of cultured hAT-MSCs. hAT-MSCs are stained positive for CD73, CD90 and CD105 but negative for CD31, CD34 and CD45. Histograms represent the relative number of cells versus fluorescent intensity. (D) hAT-MSCs were differentiated into adipocyte, osteocyte and chondrocyte cells. Neutral lipid vacuoles are indicated by adipogenesis staining by Oil Red O. The accumulation of alkaline phosphatase and calcium oxalates, shown by Alizarin Red staining, demonstrates differentiation into the osteoblast lineage. Chondrogenic differentiation is indicated by Alcian blue staining to detect type II collagen. Scale bar, 100 μm.

BTICs-like cells from three types of primary brain tumors were cultured and established. To define the BTICs, we confirmed sphere formation and stem cell marker expression *in vitro* (Fig 1A and S1 Fig) and tumorigenic capability *in vivo* (tumor formation in 6 of 6 mice for medulloblastoma-BTICs, 6 of 6 mice for AT/RT-BTICs and 6 of 6 mice for glioblastoma-BTICs after 2 months, Fig 1B).

The hAT-MSCs were isolated and characterized. The isolated cells showed fibroblast-like morphology, which is a characteristic of MSCs. To further characterize the hAT-MSC population, the cells were analyzed for the expression of cell membrane protein markers using flow cytometry analysis. The expression of CD73, CD90 and CD105, which are generally considered markers of MSCs, was positive, whereas that of the hematopoietic markers CD34 and CD45 was negative (Fig 1C). The absence of CD31 expression excluded the possibility of endothelial cell contamination. Oil Red O staining showed that the hAT-MSCs formed intracellular lipid globules in adipogenic medium, Alizarin Red staining showed that they generated mineralized bone nodules in osteogenic medium, and Alcian blue staining showed that they aggregated into spherical masses in chondrogenic differentiation medium (Fig 1D).

### Migration of hAT-MSCs toward BTICs in vitro

**Fig 2:**
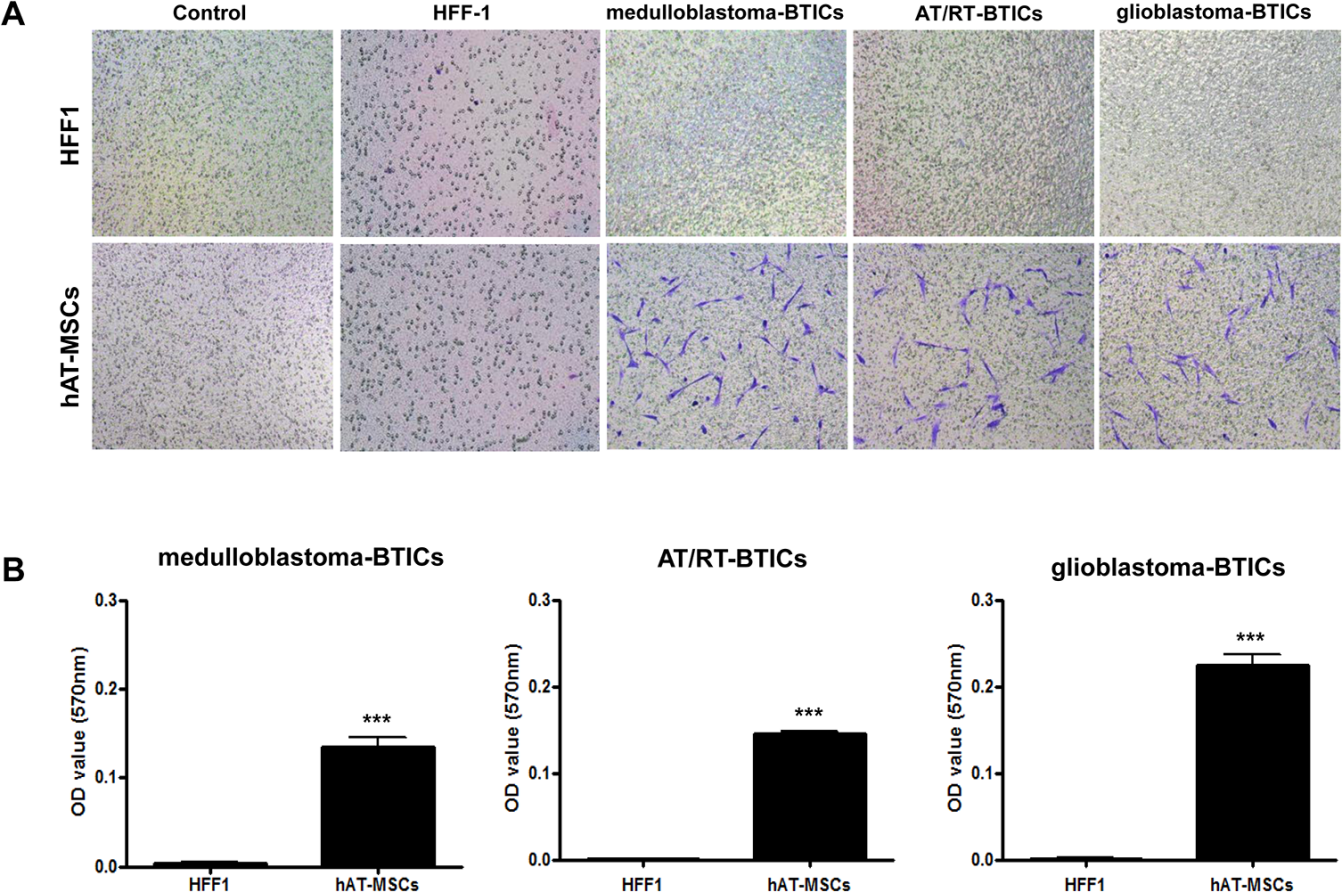
Migratory ability of human adipose tissue-derived mesenchymal stem cells (hAT-MSCs) toward brain tumor-initiating cells (BTICs) *in vitro*. (A) The migration ability of hAT-MSCs was assessed and compared with that of HFF1 cells. Representative images of stained filters show migrated hAT-MSCs toward medulloblastoma-BTICs, atypical teratoid/rhabdoid tumors (AT/RT)-BTICs and glioblastoma-BTICs. hAT-MSCs exhibited greater migratory abilities toward BTICs compared with HFF1 cells and hAT-MSCs did not migrate toward HFF1; ×40 magnification. (B) Migration was quantified based on the OD value after dissolving the stained cells. The data shown are averages of quadruplicate wells, and bars represent ± SD. ***Significant difference from control (P < 0.001).

The migratory capacity of hAT-MSCs toward BTICs from three different tumors (medulloblastoma, AT/RT and glioblastoma) was evaluated using a modified transwell assay. Compared with the HFF1 cells, the hAT-MSCs had greater migratory ability toward BTICs (HFF1 versus hAT-MSCs: 0.003 ± 0.001 versus 0.135 ± 0.011, p < 0.0001 for medulloblastoma; 0.001 ± 0.001 versus 0.146 ± 0.007, p < 0.0001 for AT/RT; 0.002 ± 0.001 versus 0.225 ± 0.037, p < 0.0001 for glioblastoma, Fig 2). Neither HFF1 nor hAT-MSCs migrated in the absence of BTICs. hAT-MSCs did not migrate toward HFF1.

### Migration of hAT-MSCs toward BTICs in vivo

**Fig 3:**
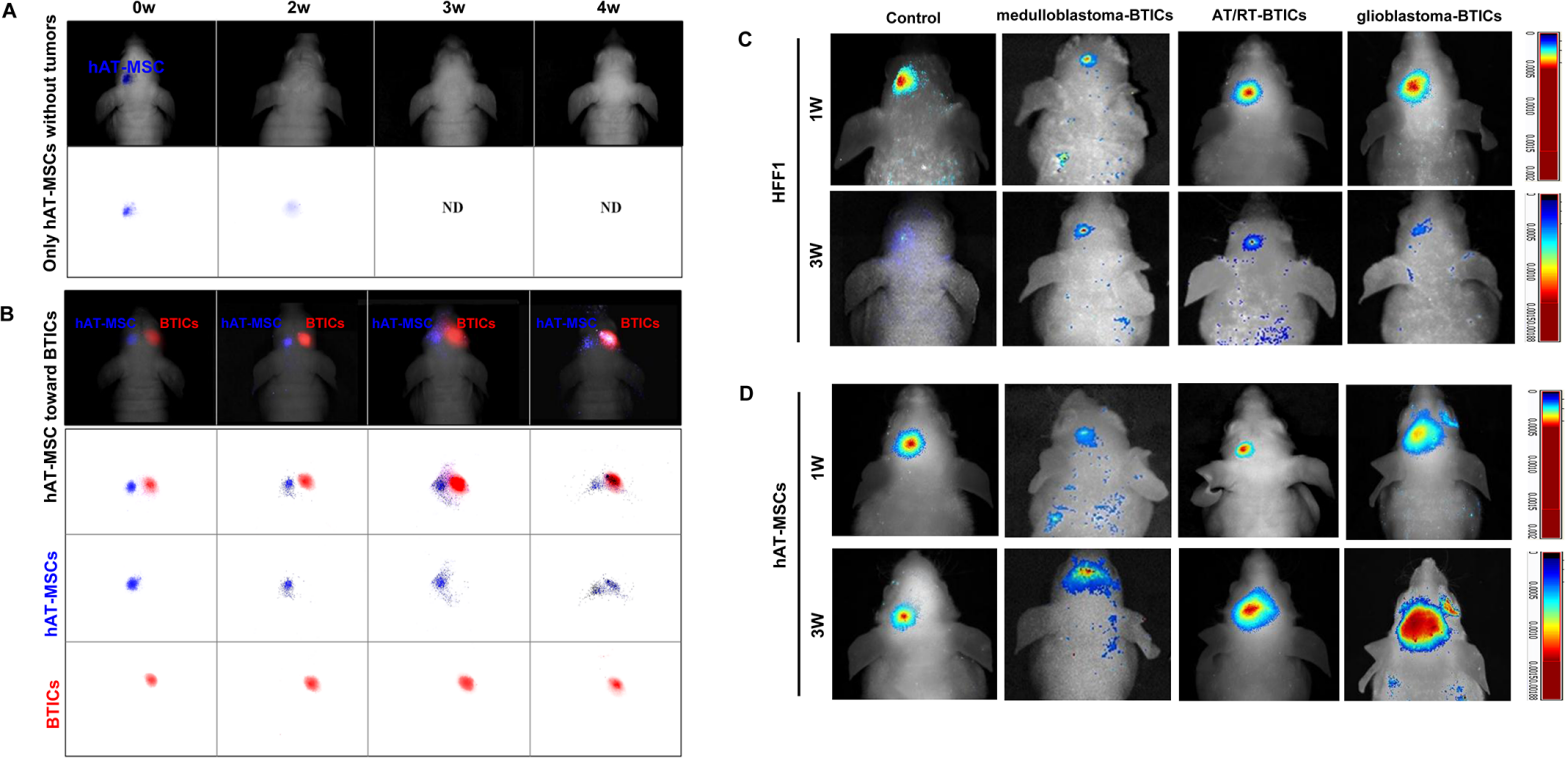
Migratory ability of human adipose tissue-derived mesenchymal stem cells (hAT-MSCs) toward brain tumor-initiating cells (BTICs) *in vivo*. Fluorescence images of the brains were taken at the indicated times. (A) In the absence of tumor, the fluorescent signals of hAT-MSCs (blue) are weakened at 2 weeks and are not detectable at 3 weeks. (B) *In vivo* sequential tracking was performed by injecting both NEO-LIVE 675-labeled BTICs (red) and NEO-LIVE 797-labeled hAT-MSCs (blue). hAT-MSCs gradually migrate toward the tumor site, and strong fluorescent signals are observed at 4 weeks post-injection at the tumor site. (C) Mice were injected the NEO-LIVE 797-labeled hAT-MSCs or HFF1. Representative *in vivo* fluorescence images show that the HFF1 signals are decreased or fade out. (D) On the contrary, the hAT-MSC signals are widened at all BTIC-derived mouse brain tumor sites at 3 weeks post-injection when compared with HFF1 cells. The color bar indicates radiant efficiency.

At first, we evaluated the *in vivo* distribution of the hAT-MSCs. In the absence of tumor implantation, signals of hAT-MSCs began to disappear after 2 weeks and were undetectable after 3 weeks (Fig 3A). When we labeled both hAT-MSCs and BTICs, the migration of hAT-MSCs toward BTICs was clearly visualized on *in vivo* imaging (Fig 3B).

Next, we compared migratory capacity of hAT-MSCs with that of control cells (HFF-1). The signals of HFF1 were very weak in control mice and localized only at the injection site of BTICs at 3 weeks (Fig 3C). Meanwhile, the fluorescent signals of the hAT-MSCs preferentially spread toward BTIC-derived tumor site in all tumor-bearing mice after 3 weeks (Fig 3D).

### Expression of cyto-chemokine receptors on hAT-MSCs

**Fig 4:**
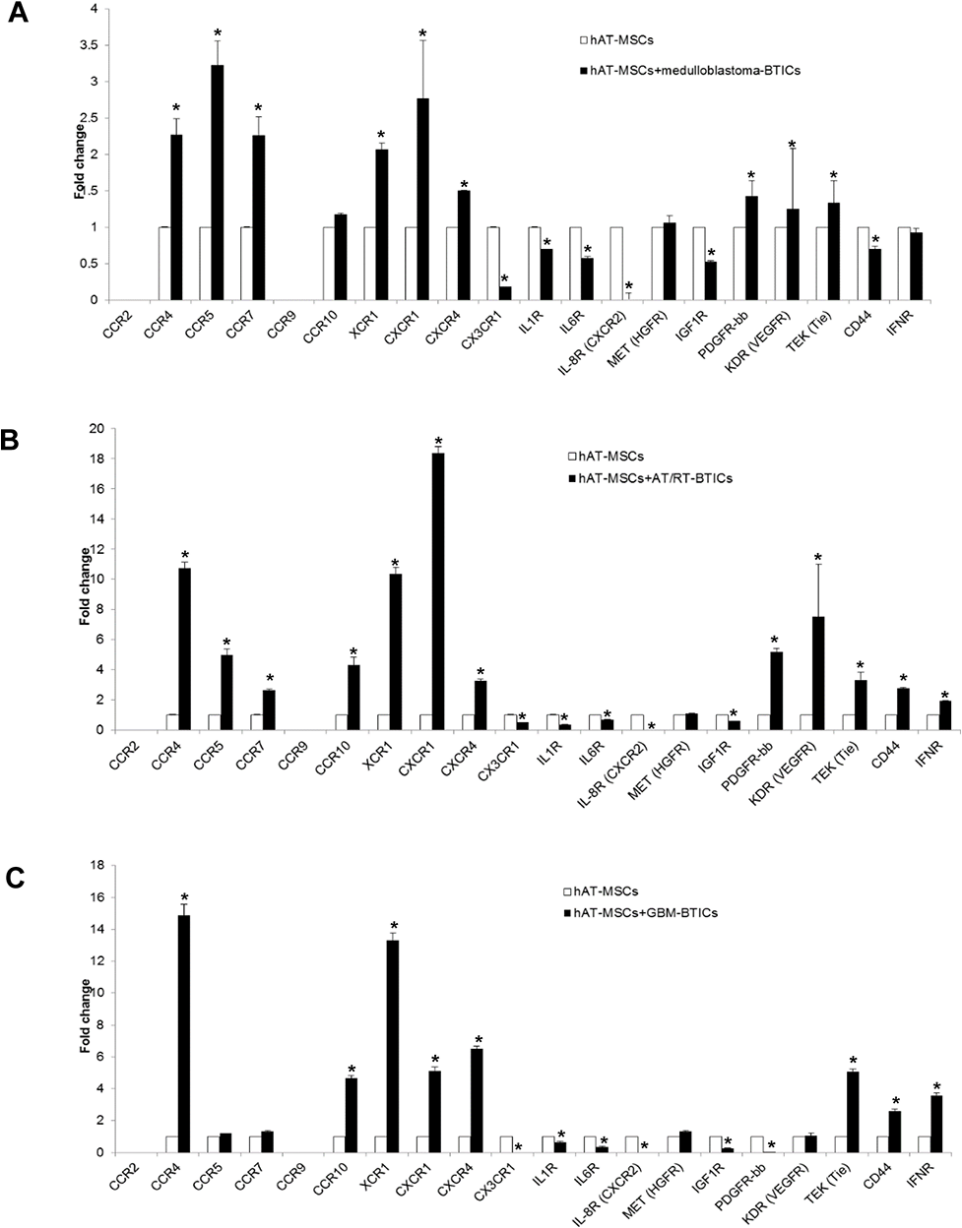
mRNA expression of cyto-chemokine receptors in human adipose tissue-derived mesenchymal stem cells (hAT-MSCs) after co-culture with brain tumor-initiating cells (BTICs). Real-time Quantitative Polymerase Chain Reaction (RT-qPCR) analysis of cyto-chemokine receptors after co-culture of (A) medulloblastoma-BTICs, (B) atypical teratoid/rhabdoid tumors (AT/RT)-BTICs and (C) glioblastoma-BTICs. The mRNA level of each cytokine receptor was normalized to the level of GAPDH. All data are representative of three independent experiments, and each value represents the mean ± SD. *Significant difference from control (P < 0.05).

We measured the mRNA expression patterns of cyto-chemokine receptors on hAT-MSCs after co-culture with BTICs and compared them to those of hAT-MSCs cultured alone. The analyses showed that the cytokine receptors were differentially expressed depending on the hAT-MSC-BTIC co-culture. When hAT-MSCs were co-cultured with medulloblastoma-BTICs, the expression levels of CCR4, CCR5, CCR7, XCR1, CXCR1, CXCR4, PDGFR-bb, KDR and TEK were increased, while the levels of CX3CR1, IL1R, IL6R, IL8R, IGF1R and CD44 were decreased (p<0.05, Fig 4A, S3 Table). For AT/RT-BTICs, the levels of CCR4, CCR5, CCR7, CCR10, XCR1, CXCR1, CXCR4, PDGFR-bb, KDR, TEK, CD44 and IFNR were increased, but the those of CX3CR1, IL1R, IL6R, IL8R and IGF1R were decreased (p<0.05, Fig 4B). For glioblastoma-BTICs, the levels of CCR4, CCR10, XCR1, CXCR1, CXCR4, TEK, CD44 and IFNR were increased, but those of CX3CR1, IL1R, IL6R, IL8R, IGF1R and PDGFR-bb were decreased (p<0.05, Fig 4C). In summary, these results suggest that the CCR4, CCR5, CCR7, CCR10, XCR1, CXCR1, CXCR4, PDGFR-bb, KDR, TEK, CD44, IFNR, CX3CR1, IL1R, IL6R, IL8R and IGF1R receptors on hAT-MSCs may be more functional in their crosstalk and may be the cardinal cytokines in the migration of hAT-MSCs toward BTICs.

### Cyto-chemokine profiling

The previous experiments suggest that hAT-MSC receptors (CCR4, CCR5, CCR7, CCR10, XCR1, CXCR1, CXCR4, PDGFR-bb, KDR, TEK, CD44, IFNR, CX3CR1, IL1R, IL6R, IL8R and IGF1R) might play major roles in the crosstalk of BTIC cyto-chemokine ligands. We further evaluated the differential secretion profiles of these cytokines in migration of hAT-MSCs toward BTIC-derived tumors.

Co-culture of BTICs and hAT-MSCs showed different expression levels of several cyto-chemokines compared with co-culture of BTICs and HFF1: SDF-1, RANTES, IL-6 and IL-8 in medulloblastoma-BTICs, SDF-1, RANTES, IL-6 and IL-8 in AT/RT-BTICs and SDF-1, IL-6, IL-8 and IGF-1 in glioblastoma-BTICs (Table 1). Interestingly, the increased level of SDF-1 and decreased level of the immuno-modulating cytokines IL-6 and IL-8 were commonly observed in all BTICs co-cultured with hAT-MSCs. RANTES was increased in medulloblastoma-BTICs and AT/RT-BTICs, but not in glioblastoma. IGF-1 was increased only in glioblastoma-BTICs co-cultured with hAT-MSCs.

**Table 1:** Induced cytokine levels in the BTICs after co-cultured with HFF1 or hAT-MSCs (pg/ml)*.

BTICs Ligands	Medulloblastoma-BTICs + HFF1	Medulloblastoma-BTICs + hAT-MSCs	AT/RT-BTICs + HFF1	AT/RT-BTICs + hAT-MSCs	Glioblastoma-BTICs + HFF1	Glioblastoma-BTICs + hAT-MSCs
MCP-1	-3.3 ± 3.8	-4.8 ± 3.4	-28.1 ± 4.6	-20.4 ± 0.01	721.9 ± 516.7	-15.0 ± 3.8
SDF-1	40.9 ± 23.3	97.9 ± 17.7	-29.6 ± 82.4	74.4 ± 4.6	32.4 ± 12.4	138.4 ± 40.7
RANTES	2.7 ± 40.0	130.0 ± 108.8	64.2 ± 15.9	107.4 ± 70.5	104.6 ± 7.2	41.7 ± 57.1
IL-6	270.4 ± 221.2	-7372.2 ± 4280.7	144.4 ± 58.1	-7383.1 ± 5350.2	249.8 ± 98.5	-1908.4 ± 4577.3
IL-8	5631.6 ± 3985.2	-432.3 ± 2740.2	2627.7 ± 58.1	-6381.1 ± 5350.2	7684.3 ± 98.5	-5069.3 ± 4577.3
IGF-1	-477.5 ± 355.5	-343.5 ± 349.0	-1251.5 ± 614.1	-2307.0 ± 289.9	-786.0 ± 840.0	2090.5 ± 117.4
PDGF-bb	6.1 ± 0.001	3.0 ± 0.9	96.4 ± 52.8	22.6 ± 7.0	8.9 ± 1.9	2.4 ± 1.7
VEGF	100.4 ± 8.2	-1766.1 ± 708.1	241.3 ± 99.0	-1762.8 ± 897.5	115.5 ± 68.3	-1908.4 ± 838.3
Ang-1	2645.4 ± 77.1	189.7 ± 147.4	58.2 ± 15.9	91.3 ± 77.9	2852.8 ± 62.8	199.8 ± 121.4

BTICs: brain tumor initiating cells, HFF1: human foreskin fibroblast, hAT-MSCs: human adipose-derived mesenchymal stem cells, AT/RT: atypical teratoid rhabdoid tumor, MCP-1: monocyte chemoattractant protein 1, SDF-1: stromal cell-derived factor 1, RANTES: regulated on activation, normal T cell expressed and secreted, IL-6: interleukin-6 ligand, IL-8: interleukin-8, IGF-1: insulin-like growth factor 1ligand, PDGF-bb: platelet-derived growth factor, VEGF: vascular endothelial growth factor, Ang-1: angiopoietin1

*[induced cytokine level in the BTICs with HFF1] = [total cytokine level in the BTICs with HFF1]-[cytokine level in the HFF1 only]

[induced cytokine level in the BTICs with hAT-MSCs] = [total cytokine level in the BTICs with hAT-MSCs]-[cytokine level in the hAT-MSCs only]

### Protein expression of SDF-1, RANTES, IGF1, IL-6 and IL-8 in BTICs co-cultured with hAT-MSCs or HFF1

**Fig 5:**
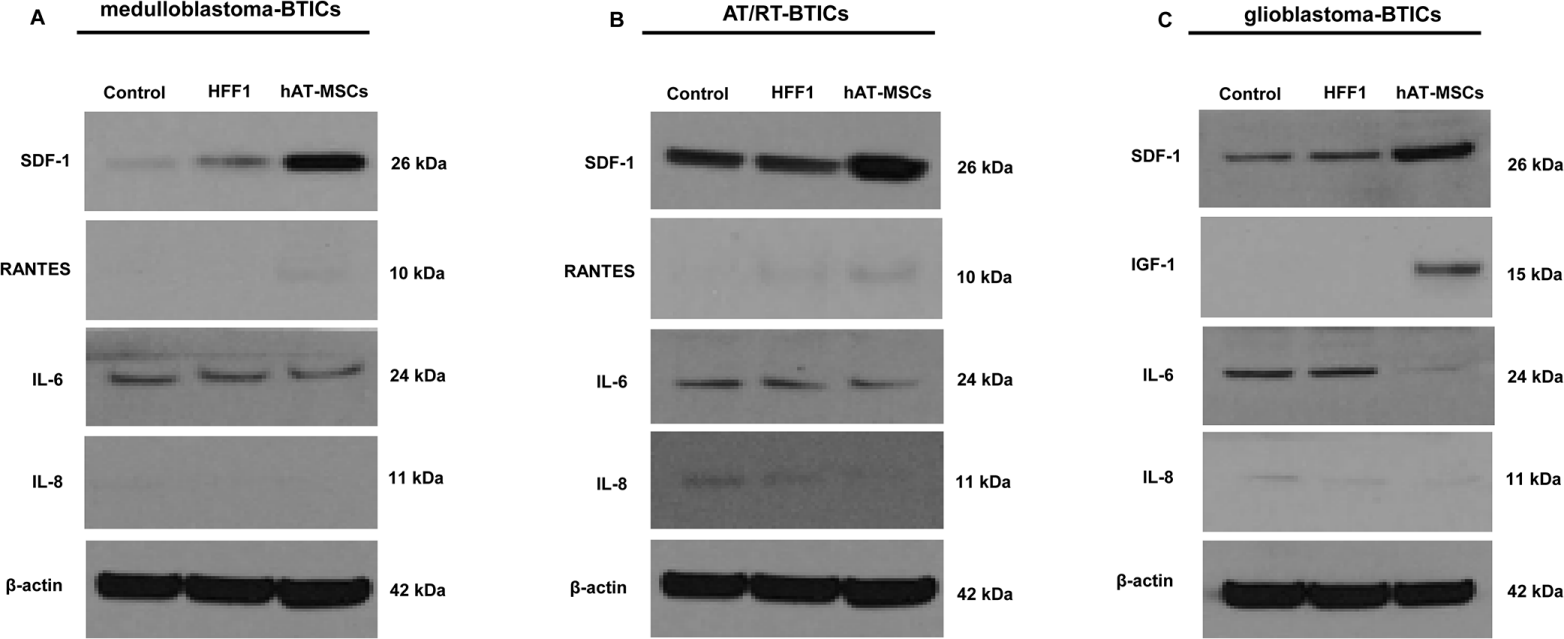
Cyto-chemokine ligand protein expression in brain tumor-initiating cells (BTICs) after co-culture with human adipose tissue-derived mesenchymal stem cells (hAT-MSCs) or HFF1 cells. Western blot analysis shows the increased expression of SDF-1 and decreased expression of IL-8 in all BTICs co-cultured with hAT-MSCs. (A and B) In medulloblastoma-BTICs and atypical teratoid/rhabdoid tumors (AT/RT)-BTICs, the expression of RANTES is increased, but that of IL-8 is not changed. (C) In glioblastoma-BTICs co-cultured with hAT-MSCs, the expression of IGF-1 is higher but that of IL-6 is lower. All data are representative of three independent experiments.

We performed western blotting to confirm the results of cyto-chemokine analysis at the protein level. The protein expression of SDF-1 was higher in all BTICs and that of RANTES was higher in medulloblastoma and AT/RT-BTICs (Fig 5A and 5B). Interestingly, IGF-1 was elevated only in glioblastoma-BTICs (Fig 5C). Furthermore, the protein level of IL-8 was decreased in all BTICs, and the level of IL-6 was decreased in glioblastoma-BTICs when co-cultured with hAT-MSCs (Fig 5).

### Inhibition of migration of hAT-MSCs toward BTICs by cytokine receptors knock down

**Fig 6:**
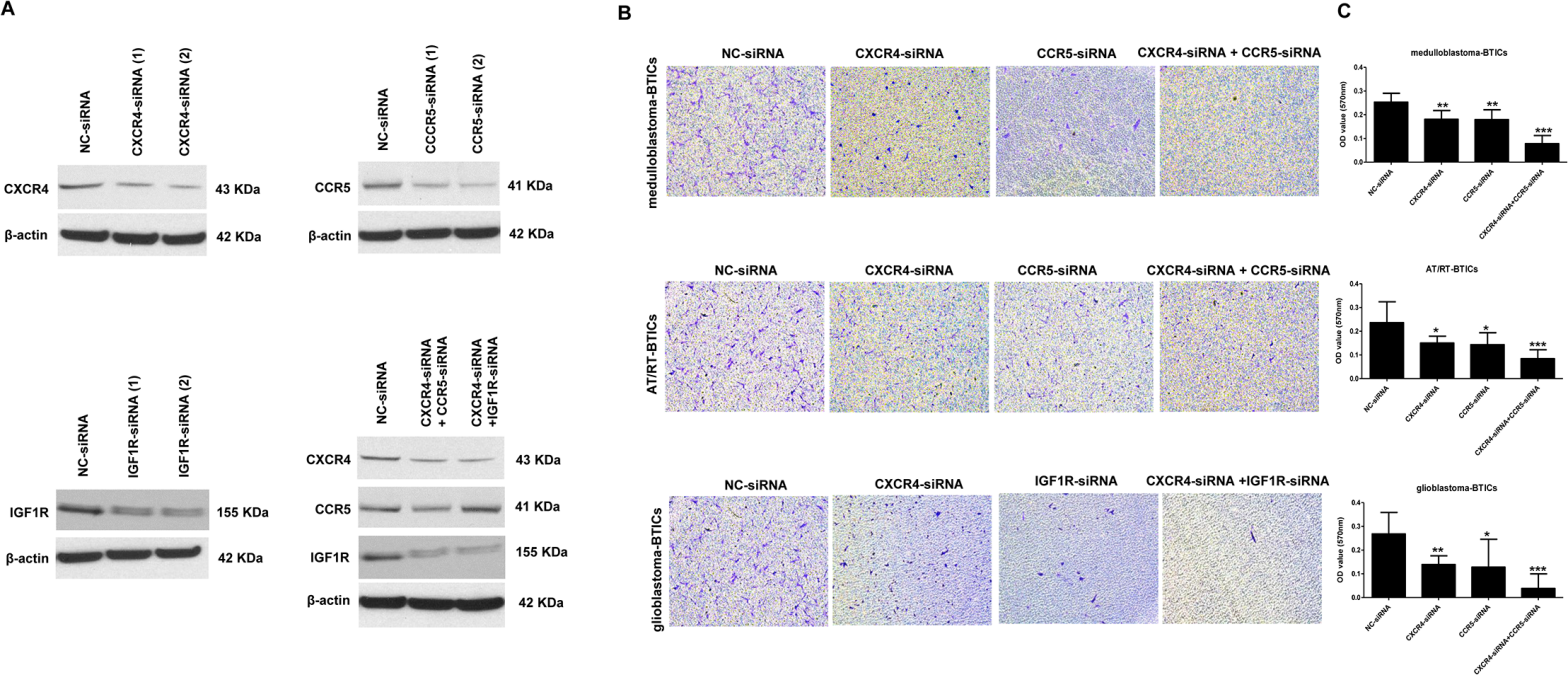
Knock down of cytokine receptors on hAT-MSCs. After siRNAs treatment to each cytokine receptors on hAT-MSCs, (A) the protein expressions were confirmed in hAT-MSCs by western blot and (B) the migratory ability toward BTICs is assessed using trans-well assay. (C) The quantified results show inhibition of migration by selective knock down of cytokine receptors. ×50 magnification. All data are representative of three independent experiments, and each value represents the mean ± SD. *P < 0.05, **P < 0.01, ***P < 0.001.

To identify the biological function of cytokine receptors, we selectively knocked down CXCR4, CCR5, IGF1R, CXCR4 + CCR5 or CXCR4 + IGF1R on hAT-MSCs using siRNA. Knock down of cytokines by siRNA in hAT-MSCs was efficient (Fig 6A) and reduced the migration of hAT-MSCs toward all BTICs (Fig 6B). Interestingly, double knock down of CXCR4 + CCR5 was more effective in decreasing the migration of hAT-MSCs toward medulloblastoma-BTICs or AT/RT-BTICs (Fig 6B and 6C and S4 Table). Synergistic inhibition of migration of hAT-MSCs toward glioblastoma-BTICs was also confirmed after double knock down of CXCR4 + IGF1R (Fig 6B and 6C and S4 Table).

## Discussion

The discovery of NSCs has resulted in a paradigm shift in brain tumor research. The biology of NSCs has been pursued as evil twins [3] and magic bullets [16]. ‘Evil twins’ refers to TICs. BTICs are a rare population of undifferentiated cells with NSC-like properties and are important for understanding the origin and maintenance of brain tumors. ‘Magic bullets’ refer to the tumor-targeting capacity of NSCs. NSCs can be used as a potential cell-based vehicle for gene therapy.

NSCs and MSCs are known to exhibit migratory abilities toward brain tumors [8–14, 17–19]. However, to effectively attack tumors and prevent relapses, it is important to target BTICs, which are responsible for tumor initiation and chemo/radiation therapy resistance [20]. In the present study, we demonstrated the migratory ability of hAT-MSCs toward BTICs through signaling involving certain cytokines in the tumor microenvironment.

*In vitro* and *in vivo* experimental data showed that hAT-MSCs possess migratory abilities to target BTICs isolated from three different types of tumors, including medulloblastoma, AT/RT and glioblastoma. Furthermore, this study validated the safety of the stem cell-based therapy; when BTICs were absent in the brain, hAT-MSCs did not show their targeting abilities and disappeared with time. These findings supported the safety of hAT-MSC-based therapy harboring the potential for autologous transplantation and lack of mesenchymal differentiation in the central nervous system [9].

Many different cytokines [21–23], chemokines [23–26] and growth factors [23, 27] have been suggested to regulate the migration of stem/progenitor cells in tumor microenvironments. However, little is known about the crosstalk between MSCs and BTICs.

At first, we evaluated the mRNA expression of cyto-chemokine receptors in hAT-MSCs after co-cultures with BTICs. From this result, we found that diverse types of receptors, including CCR4, CCR5, CCR7, CCR10, XCR1, CXCR1, CXCR4, PDGFR-bb, KDR, TEK, CD44, IFNR, CX3CR1, IL1R, IL6R, IL8R and IGF1R, were involved in crosstalk between hAT-MSCs and BTICs. Next, we performed ELISA cyto-chemokine analysis to identify putatively important ligands of BTICs. We observed different expression level of SDF-1, RANTES, IL-6 and IL-8 in medulloblastoma-BTICs, SDF-1, RANTES, IL-6 and IL-8 in AT/RT-BTICs and SDF-1, IL-6, IL-8 and IGF-1 in glioblastoma-BTICs. Then, we performed western blot analysis to determine whether our findings were consistent at the protein level. Lastly, we conducted the knock down assay to find out which cytokine receptors regulate the migration ability of hAT-MSCs toward BTICs. Based on our experiments, we indicated that different types of cyto-chemokines are involved in the crosstalk between hAT-MSCs and BTICs (CXCR4/SDF-1, CCR5/RANTES, IL6R/IL-6 and IL8R/IL8 in medulloblastoma-BTICs and AT/RT-BTICs; CXCR4/SDF-1, IL6R/IL-6, IL8R/IL-8 and IGF1R/IGF-1 in glioblastoma-BTICs).

SDF-1, which binds to its receptor CXCR4, plays an important and unique role in the regulation of stem/progenitor cell trafficking [28]. RANTES enhances the motility, invasion and metastasis of breast cancer cells [29], and IGF-1 enhances extracellular matrix production [30]. Interestingly, we found that IL-6 and IL-8 were significantly reduced when BTICs were co-cultured with hAT-MSCs. IL-6 is known to participate in the immune response or tumor metastasis, and IL-8 contributes to cancer progression through its potential functions as a mitogenic, motogenic and angiogenic factor [31]. These results might suggest the immunomodulatroy properties of hAT-MSCs. Although our results showed that the cytokine receptors of hAT-MSCs were related to BTIC ligands, it is still not clear whether BTICs send signals to hAT-MSCs or if hAT-MSCs excrete cyto-chemokines that are attracted by BTICs. Recent studies reported that exosomes secreted by cancer cells may play an important role in cancer progression, in turn, exosomes secreted by stromal cells in the tumor microenvironment may contribute to cancer progression through the transmission of their cargo to cancer cells [32,33]. MSCs are prolific producers of exosomes and exosomes have also been examined for their therapeutic potential in cancers. Exosomes include not only miRNAs but also many types of mRNAs, proteins, and cytokines, it is possible that certain proteins function additively [34–36]. Therefore, further *in vivo* studies on receptor/ligand interactions and the specific factors responsible for this targeted tropism are required to fully understand the crosstalk between hAT-MSCs and BTICs.

In conclusion, we demonstrated that hAT-MSCs could target BTICs, suggesting the potential of hAT-MSC-based gene therapy. With the elimination of BTICs, which allow cancer to renew and relapse, better prognoses of various types of brain tumors would be expected. Various cytokines, including CXCR4/SDF-1, CCR5/RANTES, IGF1R/IGF-1, IL6R/IL-6 and IL8R/IL-8, play key roles in the tropism and immune modulation of hAT-MSCs toward BTICs. Understanding the crosstalk between hAT-MSCs and BTICs could lead to more effective therapeutic strategies for curing brain tumors.

## Supporting Information

S1 Fig Tumor spheres show BTIC characteristics. (A and B) Confocal microscopic analysis of tumor spheres stained with Sox2 and the enlarged photograph. Scale bar, 50 μm.
(TIF)

S1 Table Summary of patient population. (DOC)

S2 Table Cytokine receptors, taqman assay ID list, ligands and analysis kit. (DOC)

S3 Table mRNA expression patterns of cyto-chemokine receptors in hAT-MSCs. (DOC)

S4 Table Quantification of migratory ability of hAT-MSCs after cytokine receptors knockdown. (DOC)
