# Correction: Human Adipose Tissue-Derived Mesenchymal Stem Cells Target Brain Tumor-Initiating Cells

**DOI:** 10.1371/journal.pone.0132877

**Published:** 2015-07-28

**Authors:** Seung Ah Choi, Ji Yeoun Lee, Sung Eun Kwon, Kyu-Chang Wang, Ji Hoon Phi, Jung Won Choi, Xiong Jin, Ja Yun Lim, Hyunggee Kim, Seung-Ki Kim

The beta actin band in Fig 5B incorrectly appears as a duplicate of the beta actin band in Fig 5A. The authors have provided a corrected version of Fig 5 here, and the raw, uncropped blots are provided as Supporting Information.

**Fig 5:**
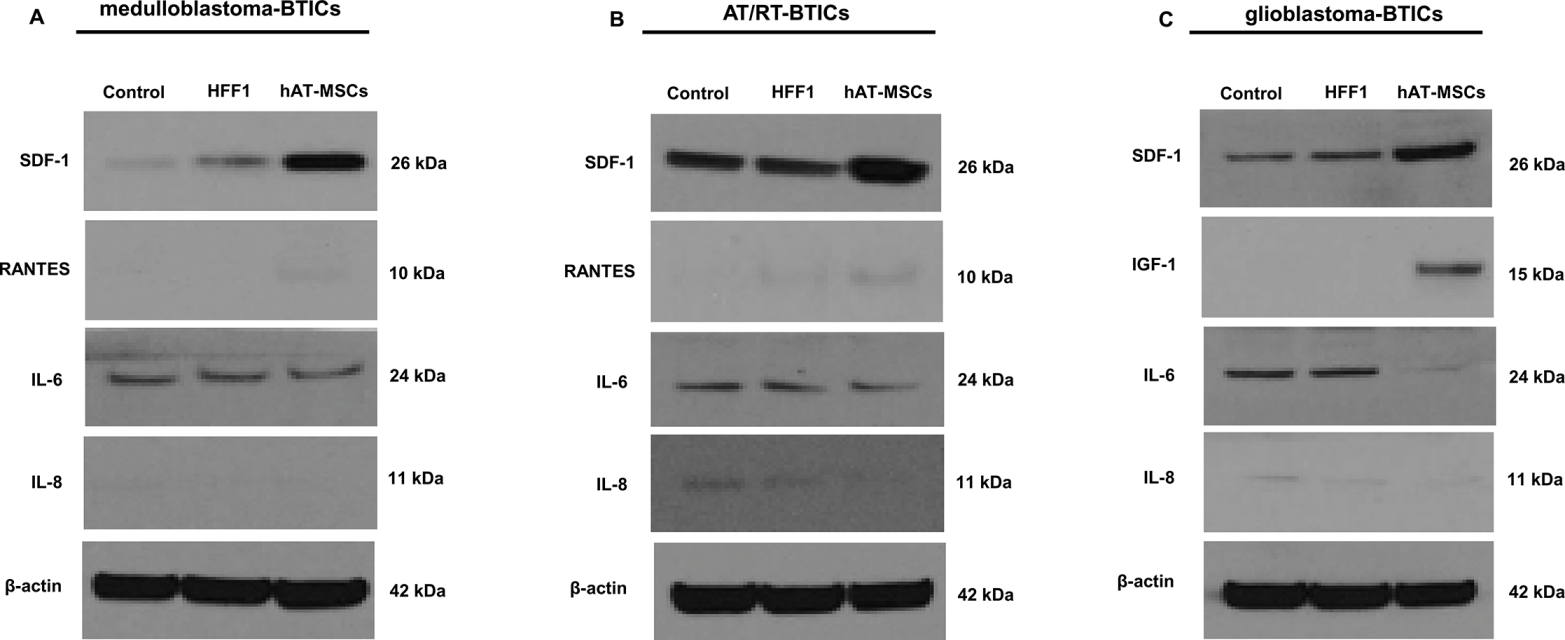
Cyto-chemokine ligand protein expression in brain tumor-initiating cells (BTICs) after co-culture with human adipose tissue-derived mesenchymal stem cells (hAT-MSCs) or HFF1 cells. Western blot analysis shows the increased expression of SDF-1 and decreased expression of IL-8 in all BTICs co-cultured with hAT-MSCs. (A and B) In medulloblastoma-BTICs and atypical teratoid/rhabdoid tumors (AT/RT)-BTICs, the expression of RANTES is increased, but that of IL-8 is not changed. (C) In glioblastoma-BTICs co-cultured with hAT-MSCs, the expression of IGF-1 is higher but that of IL-6 is lower. All data are representative of three independent experiments.

## Supporting Information

S1 File Raw, uncropped blots for Fig 5. Cyto-chemokine ligand protein expression in brain tumor-initiating cells (BTICs) after co-culture with human adipose tissue-derived mesenchymal stem cells (hAT-MSCs) or HFF1 cells. β-actin was used as protein loading control. The edges of membrane and size (kDa) were marked in blot. The proteins were loaded the marker (M), only BTICs (Control: C), BTICs co-cultured with HFF1 (H), BTICs co-cultured with hAT-MSCs (A) in the order named. In medulloblastoma-BTICs and atypical teratoid/rhabdoid tumors (AT/RT)-BTICs, the blots were arranged in β-actin, SDF-1, IL-8, IL-6 and RANTES. In glioblastoma-BTICs, the blots were arranged in β-actin, SDF-1, IL-8, IGF-1 and IL-6. The patterns of protein expression were the increased expression of SDF-1 and decreased expression of IL-8 in all BTICs co-cultured with hAT-MSCs. In medulloblastoma-BTICs and AT/RT-BTICs, the expression of RANTES is increased, but that of IL-8 is not changed. In glioblastoma-BTICs co-cultured with hAT-MSCs, the expression of IGF-1 is higher but that of IL-6 is lower.
(DOCX)
