# Supplementary figures and images for: Human Adipose Tissue-Derived Mesenchymal Stem Cells Target Brain Tumor-Initiating Cells
Source: PLoS One. 2015 Jun 15;10(6):e0129292. doi: 10.1371/journal.pone.0129292 (PMC4468214; doi:10.1371/journal.pone.0129292)

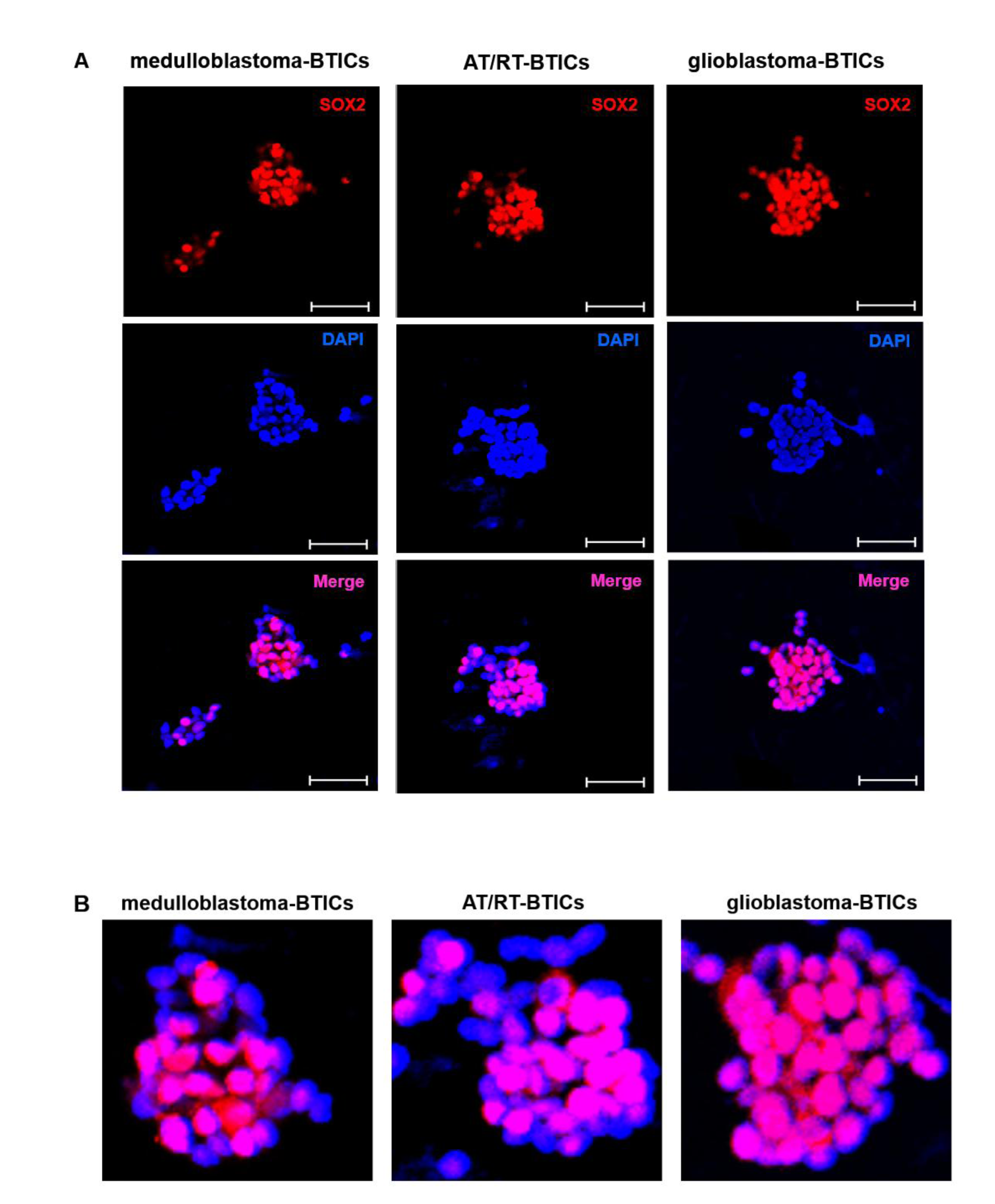

Supplement: S1 Fig — (A and B) Confocal microscopic analysis of tumor spheres stained with Sox2 and the enlarged photograph. Scale bar, 50 μm. (TIF) [file pone.0129292.s001.tif]
