# Supplementary material for: Human Adipose Tissue-Derived Mesenchymal Stem Cells Target Brain Tumor-Initiating Cells
Source: PLoS One. 2015 Jun 15;10(6):e0129292. doi: 10.1371/journal.pone.0129292 (PMC4468214; doi:10.1371/journal.pone.0129292)
Supplement: S1 Table — (DOC) [file pone.0129292.s002.doc]

**Supplementary Table S1. Summary of patient population**

| No | Pathological subtype | Age (years) | Sex | | Tumor location |
| --- | --- | --- | --- | --- | --- |
| 1 | Medulloblastoma | 9 | Male | | Cerebellum |
| 2 | Medulloblastoma | 3 | Male | | Cerebellum |
| 3 | Medulloblastoma | 12 | Male | | Cerebellum |
| 4 | AT/RT | 1 | Male | | Left cerebellopontine angle |
| 5 | AT/RT | 1 month | | Male | Cerebellum |
| 6 | Glioblastoma | 57 | Male | | Right frontal |
| 7 | Glioblastoma | 70 | Male | | Right temporal |
| 8 | Glioblastoma | 36 | Female | | Left frontal |

AT/RT: atypical teratoid/rhabdoid tumors
