# Supplementary material for: Human Adipose Tissue-Derived Mesenchymal Stem Cells Target Brain Tumor-Initiating Cells
Source: PLoS One. 2015 Jun 15;10(6):e0129292. doi: 10.1371/journal.pone.0129292 (PMC4468214; doi:10.1371/journal.pone.0129292)
Supplement: S2 Table — (DOC) [file pone.0129292.s003.doc]

**Supplementary Table S2. Cytokine receptors, taqman assay ID list, ligands and analysis kit**

| No | Cytokine receptors | Abbreviations | Taqman assay ID | Cytokine ligands | Abbreviations | Cytokine analysis kit |
| --- | --- | --- | --- | --- | --- | --- |
| 1 | CCR2 | C-C chemokine receptor type 2 | MCP-1 | Monocyte Chemoattractant Protein 1 | Hs01560352_m1 | Fluorokine® MAP Base Kits |
| 2 | CCR4 | C-C chemokine receptor type 4 | MCP-α | Monocyte Chemoattractant Protein 1α | Hs99999919_m1 | Fluorokine® MAP Base Kits |
| 3 | CCR5 | C-C chemokine receptor type 5 | RANTES | Regulated on Activation, Normal T cell Expressed and Secreted | Hs00152917_m1 | Fluorokine® MAP Base Kits |
| 4 | CCR7 | C-C chemokine receptor type 7 | CCL19 | C-C chemokine ligand type 19 | Hs01013469_m1 | Fluorokine® MAP Base Kits |
| 5 | CCR9 | C-C chemokine receptor type 9 | CCL25 | C-C chemokine ligand type 25 | Hs00246403_m1 | - |
| 6 | CCR10 | C-C chemokine receptor type 10 | CCL27 | C-C chemokine ligand type 37 | Hs00706455_s1 | - |
| 7 | XCR1 | C" sub-family of chemokine receptor | XCL1 | C" sub-family of chemokine ligand | Hs00245540_s1 | - |
| 8 | CXCR1 | C-X-C chemokine receptor type 1 | IL-8 | Interleukin-8 ligand | Hs00174146_m1 | Fluorokine® MAP Base Kits |
| 9 | CXCR4 | C-X-C chemokine receptor type 4 | SDF-1 | Stromal Cell-Derived Factor 1 | Hs00237052_m1 | SDF-1 ELISA kit |
| 10 | CX3CR1 | C-X3-C chemokine receptor type 1 | SDF-1 | Stromal Cell-Derived Factor 1 | Hs00365842_m1 | - |
| 11 | IL1R | Interleukin-1 receptor | IL-1b | Interleukin-1b ligand | Hs00991002_m1 | Fluorokine® MAP Base Kits |
| 12 | IL6R | Interleukin-6 receptor | IL-6 | Interleukin-6 ligand | Hs01075667_m1 | Fluorokine® MAP Base Kits |
| 13 | IL8R | Interleukin-8 receptor | IL-8 | Interleukin-8 ligand | Hs01011557_m1 | Fluorokine® MAP Base Kits |
| 14 | MET(HGFR) | Met proto-oncogene (Hepatocyte growth factor receptor) | HGF | Hepatocyte growth factor ligand | Hs01565584_m1 | Fluorokine® MAP Base Kits |
| 15 | IGF1R | Insulin-like growth factor 1 receptor | IGF-1 | Insulin-like growth factor 1 ligand | Hs00609566_m1 | IGF1ELISA kit |
| 16 | PDGFRbb | Platelet-derived growth factor receptor, β polypeptid | PDGF | Platelet-derived growth factor | Hs01019589_m1 | Fluorokine® MAP Human Kit |
| 17 | KDR(VEGFR2) | Kinase insert domain receptor (Vascular endothelial growth factor2) | VEGF | Vascular endothelial growth factor | Hs00911700_m1 | Fluorokine® MAP Human Kit |
| 18 | TEK(Tie) | TEK Tyrosine Kinase, Endothelial (Tyrosine Kinase With Immunoglobulin-Like And EGF-Like Domains) | Ang-1 | Angiopoietin 1 | Hs00945146_m1 | Fluorokine® MAP Human Kit |
| 19 | CD44 | CD44 antigen (homing function and Indian blood group system) | - |  | Hs00174139_m1 | - |
| 20 | IFNR | Interferon Production Regulator | - |  | Hs01066115_m1 | - |
| 21 | GAPDH | Glyceraldehyde-3-Phosphate Dehydrogenase | - |  | Hs99999905_m1 | - |
