# Supplementary material for: Human Adipose Tissue-Derived Mesenchymal Stem Cells Target Brain Tumor-Initiating Cells
Source: PLoS One. 2015 Jun 15;10(6):e0129292. doi: 10.1371/journal.pone.0129292 (PMC4468214; doi:10.1371/journal.pone.0129292)
Supplement: S3 Table — (DOC) [file pone.0129292.s004.doc]

**Supplementary Table S3 mRNA expression patterns of cyto-chemokine receptors in hAT-MSCs**

| Cytokine  receptors | hAT-MSCs only | hAT-MSCs + medulloblastoma-BTICs | hAT-MSCs + AT/RT-BTICs | hAT-MSCs + glioblastoma-BTICs |
| --- | --- | --- | --- | --- |
| CCR2 | 0.0 ± 0.0 | 0.0 ± 0.0 | 0.0 ± 0.0 | 0.0 ± 0.0 |
| CCR4 | 1.0 ± 0.01 | 2.27 ± 0.22 | 10.74 ± 0.40 | 14.88 ± 0.71 |
| CCR5 | 1.0 ± 0.01 | 3.23 ± 0.33 | 4.96 ± 0.39 | 1.19 ± 0 001 |
| CCR7 | 1.0 ± 0.007 | 2.26 ± 0.26 | 2.65 ± 0.03 | 1.32 ± 0.06 |
| CCR9 | 0.0 ± 0.0 | 0.0 ± 0.0 | 0.0 ± 0.0 | 0.0 ± 0.0 |
| CCR10 | 1.0 ± 0.01 | 1.17 ± 0.02 | 4.29 ± 0.55 | 4.66 ± 0.18 |
| XCR1 | 1.0 ± 0.10 | 2.07 ± 0.09 | 10.36 ± 0.45 | 13.30 ±0.46 |
| CXCR1 | 1.0 ± 0.001 | 2.77 ± 0.80 | 18.35 ± 0.46 | 5.12 ± 0.22 |
| CXCR4 | 1.0 ± 0.01 | 1.5 ± 0.01 | 3.28 ± 0.08 | 6.50 ± 0.17 |
| CX3CR1 | 1.0 ± 0.021 | 0.18 ± 0.001 | 0.49 ± 0.001 | 0.0 ± 0.0 |
| IL1R | 1.0 ± 0.012 | 0.70 ± 0.001 | 0.34 ± 0.01 | 0.66 ± 0.02 |
| IL6R | 1.0 ± 0.013 | 0.57 ± 0.03 | 0.69 ± 0.02 | 0.34 ± 0.05 |
| IL8R | 1.0 ± 0.003 | 0.0 ± 0.10 | 0.0 ± 0.001 | 0.0 ± 0.0 |
| MET(HGFR) | 1.0 ± 0.008 | 1.06 ± 0.11 | 1.11 ± 0.02 | 1.32 ± 0.04 |
| IGF1R | 1.0 ± 0.001 | 0.52 ± 0.02 | 0.57 ± 0.01 | 0.26 ± 0.01 |
| PDGFRbb | 1.0 ± 0.04 | 1.42 ± 0.22 | 5.20 ± 0.23 | 0.02 ± 0.001 |
| KDR(VEGFR2) | 1.0 ± 0.02 | 1.25 ± 0.83 | 7.51 ± 3.51 | 1.07 ± 0.17 |
| TEK(Tie) | 1.0 ± 0.009 | 1.33 ± 0.31 | 3.31 ± 0.51 | 5.09 ± 0.14 |
| CD44 | 1.0 ± 0.001 | 0.7 ± 0.04 | 2.75 ± 0.06 | 2.59 ± 0.13 |
| IFNR | 1.0 ± 0.101 | 0.92 ± 0.06 | 1.93 ± 0.03 | 3.58 ± 0.16 |
