# Supplementary material for: Human Adipose Tissue-Derived Mesenchymal Stem Cells Target Brain Tumor-Initiating Cells
Source: PLoS One. 2015 Jun 15;10(6):e0129292. doi: 10.1371/journal.pone.0129292 (PMC4468214; doi:10.1371/journal.pone.0129292)
Supplement: S4 Table — (DOC) [file pone.0129292.s005.doc]

**Supplementary Table S4 Quantification of migratory ability of hAT-MSCs after cytokine receptors knockdown**

| siRNA | medulloblastoma-BTICs | AT/RT-BTICs | Glioblastoma-BTICs |
| --- | --- | --- | --- |
| NC-siRNA | 0.208 ± 0.037 | 0.236 ± 0.088 | 0.268 ± 0.090 |
| CXCR4-siRNA | 0.181 ± 0.037 | 0.150 ± 0.029 | 0.139 ± 0.036 |
| CCR5-siRNA | 0.179 ± 0.041 | 0.143 ± 0.050 | - |
| IGF1R-siRNA | - | - | 0.128 ± 0.118 |
| CXCR4-siRNA+CCR5-siRNA | 0.078 ± 0.034 | 0.084 ± 0.037 | - |
| CXCR4-siRNA+IGF1R-siRNA | - | - | 0.039 ± 0.061 |

BTICs: brain tumor initiating cells, hAT-MSCs: human adipose-derived mesenchymal stem cells, AT/RT: atypical teratoid rhabdoid tumor, NC: negative control, CXCR4: C-X-C chemokine receptor type 4, CCR5: C-C chemokine receptor type 5 IGF1R: Insulin-like growth factor 1 receptor
