# Supplementary figures and images for: Correction: Human Adipose Tissue-Derived Mesenchymal Stem Cells Target Brain Tumor-Initiating Cells
Source: PLoS One. 2015 Jul 28;10(7):e0132877. doi: 10.1371/journal.pone.0132877 (PMC4517905; doi:10.1371/journal.pone.0132877)

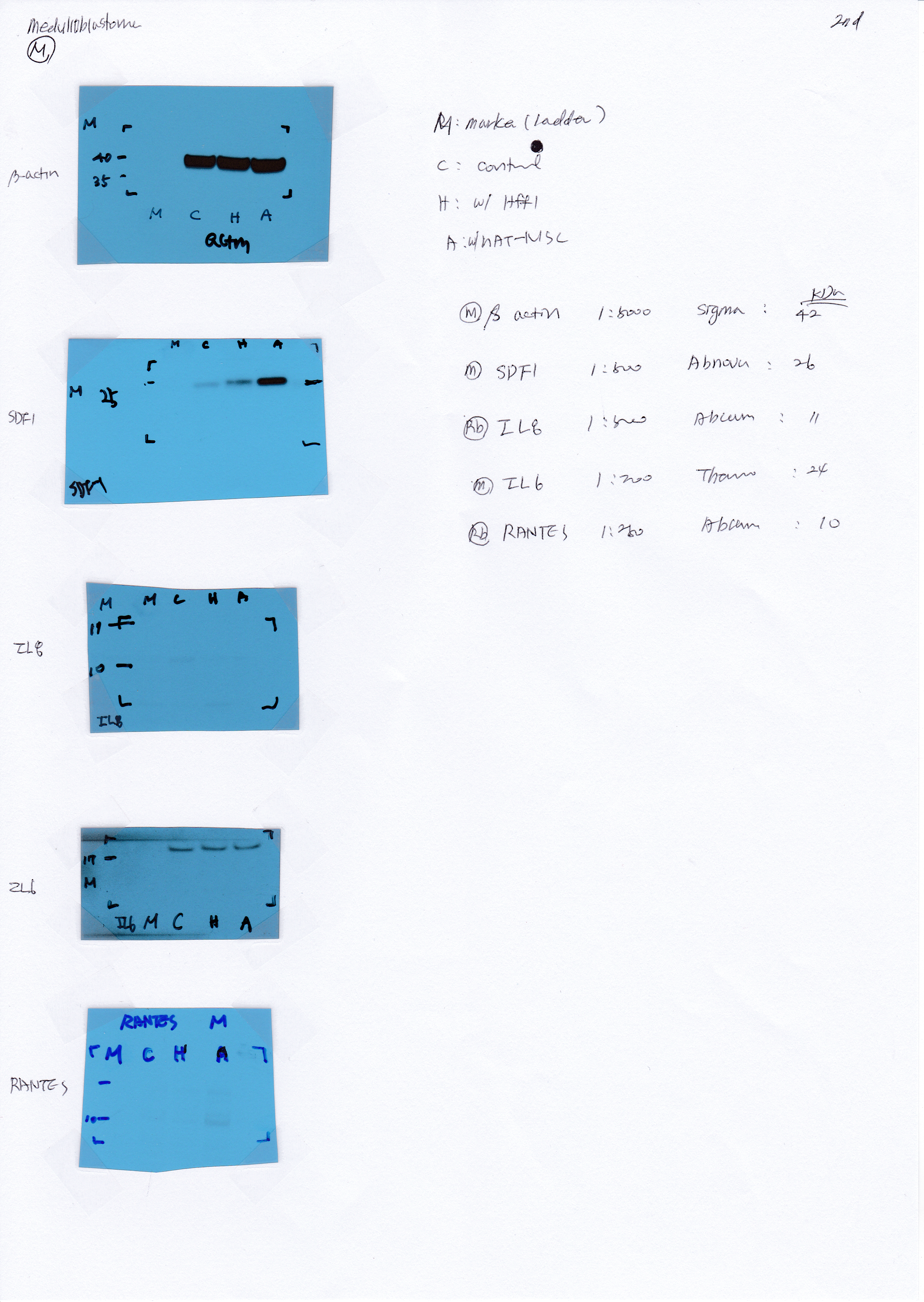


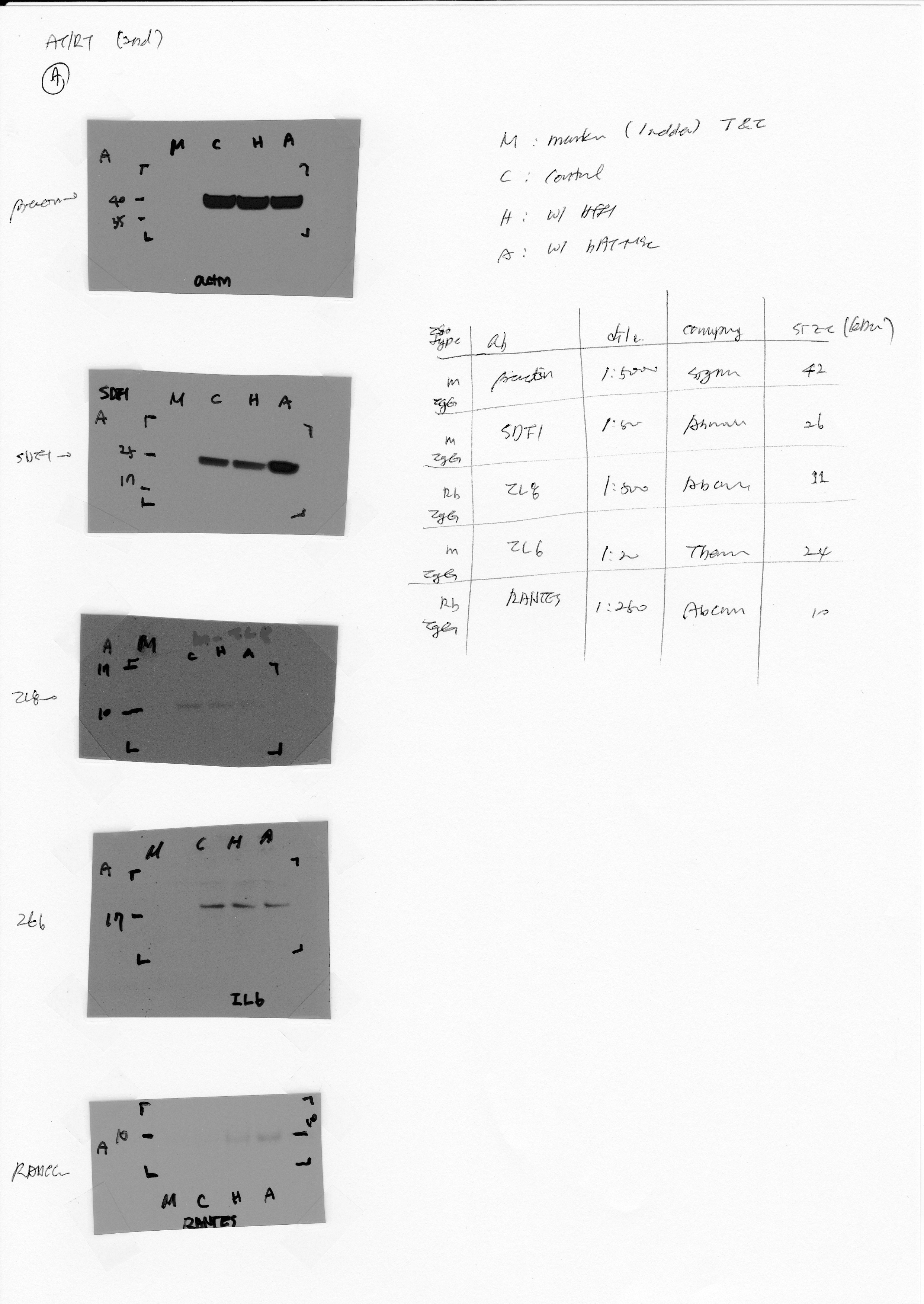


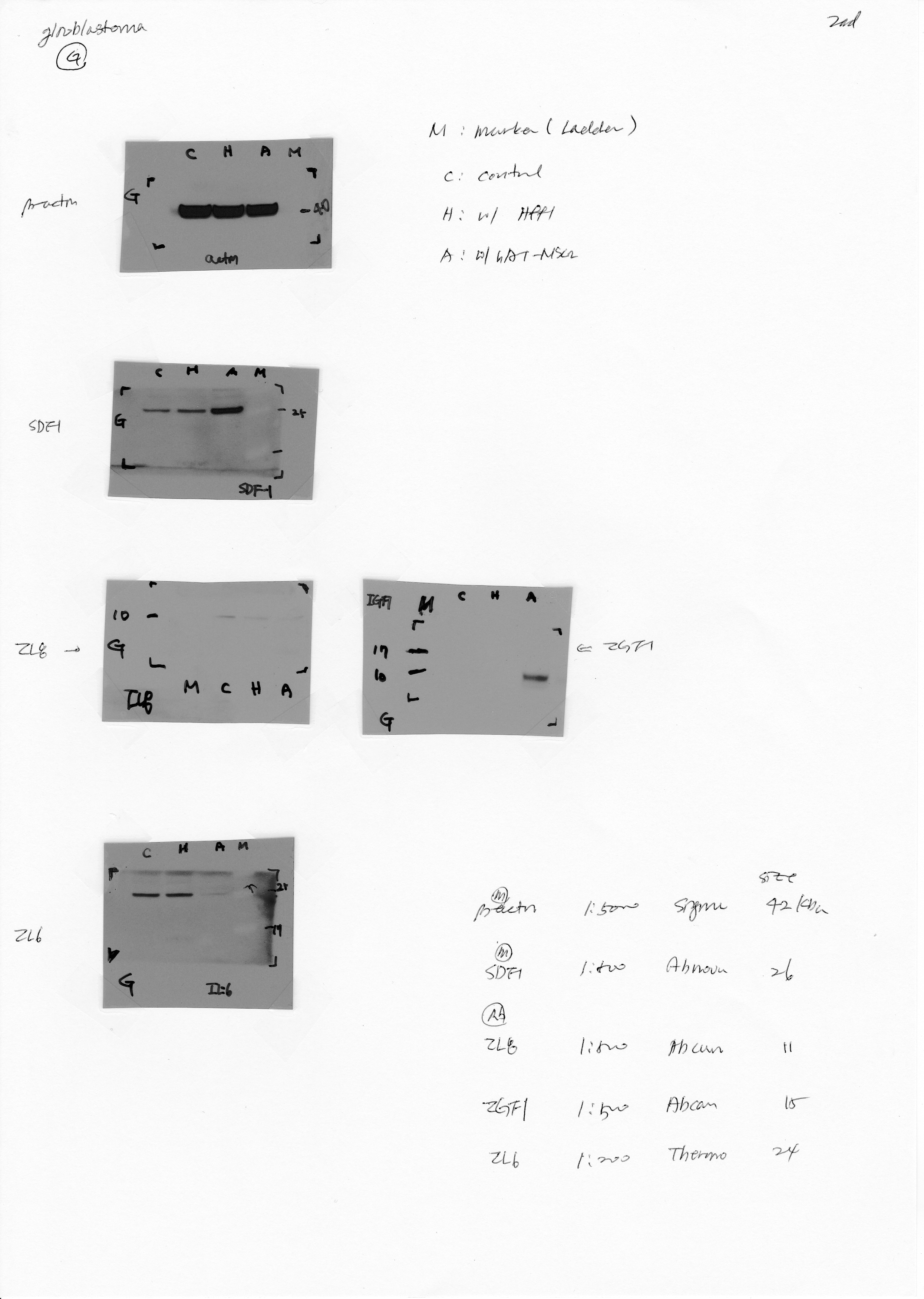


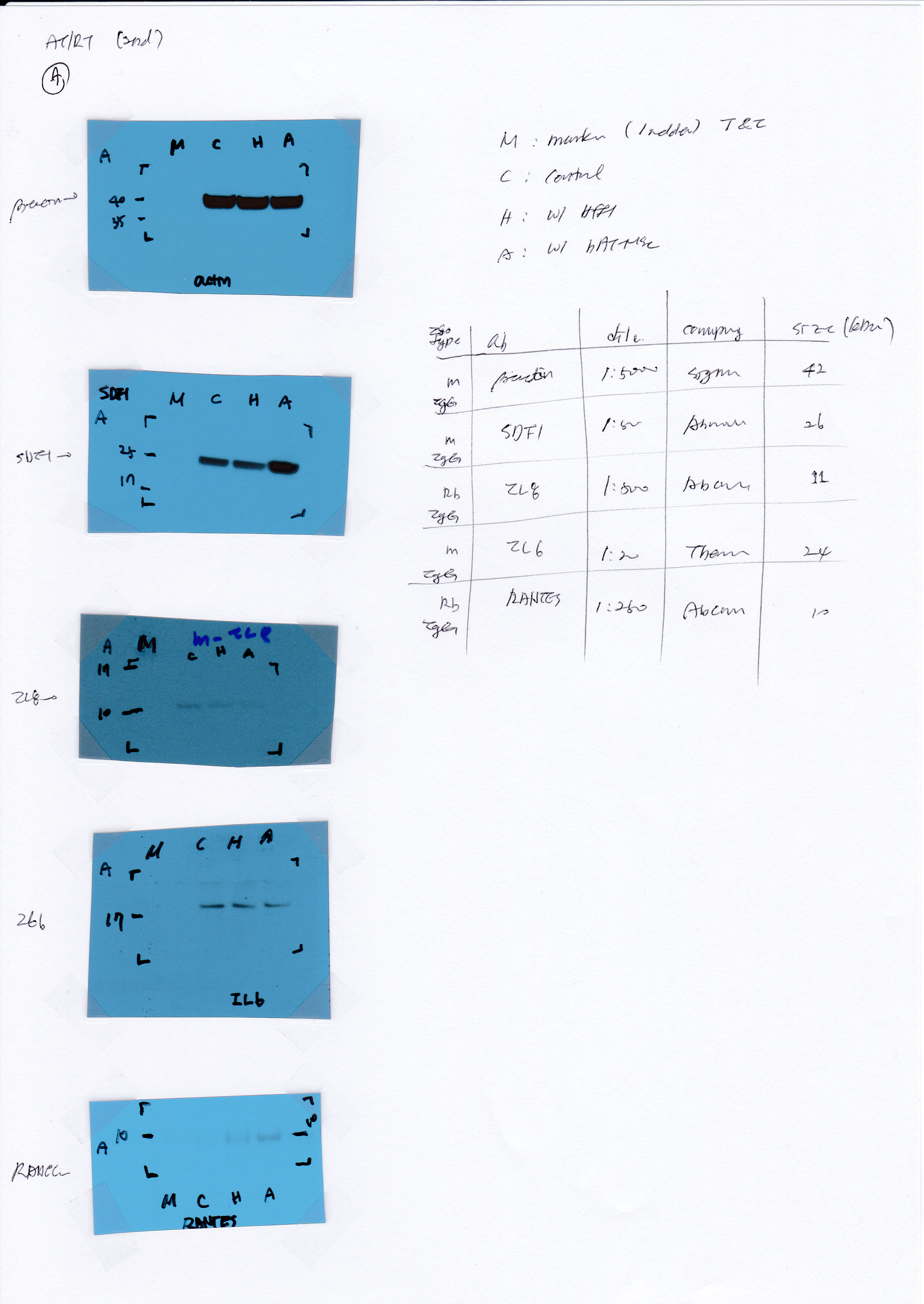


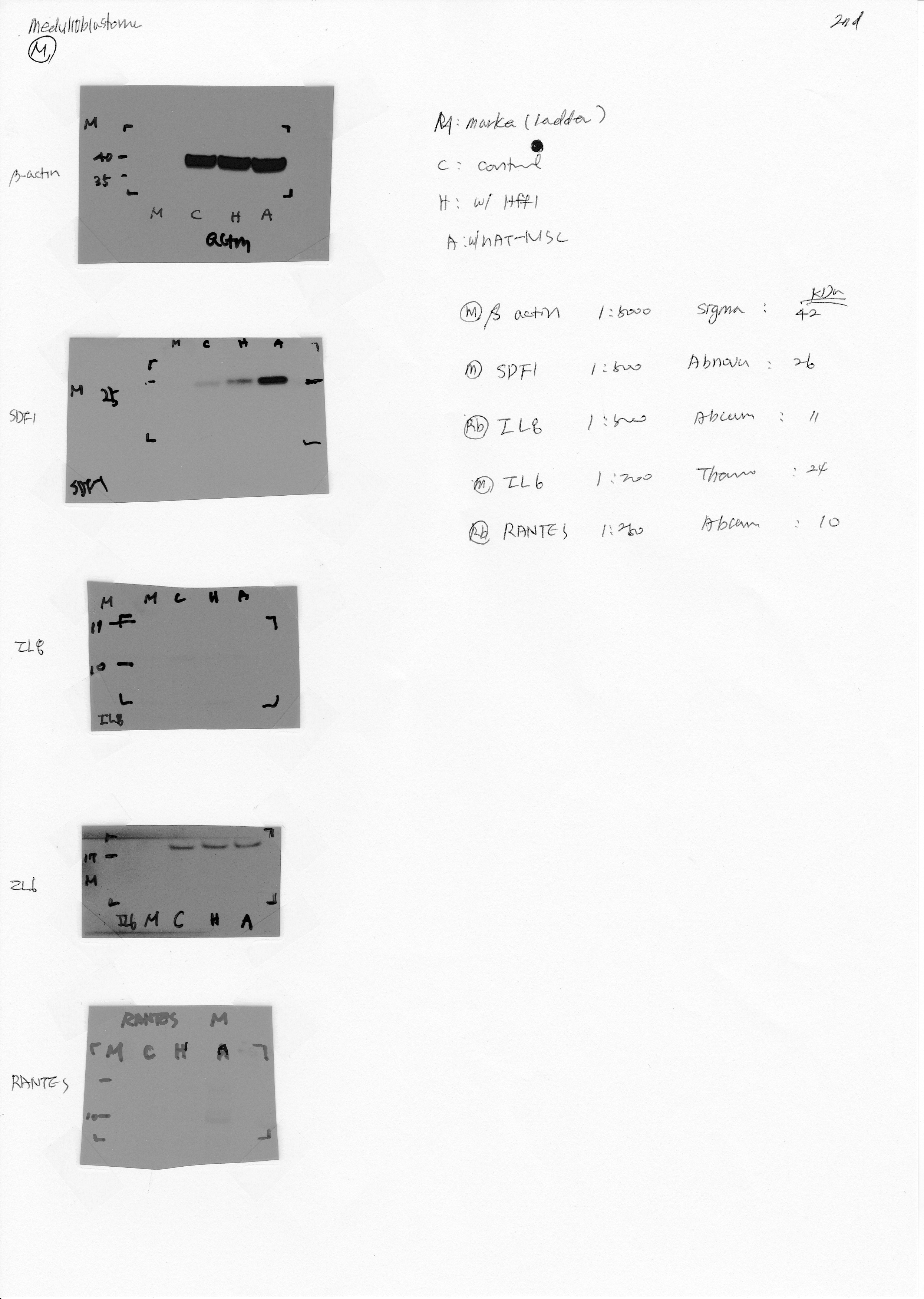


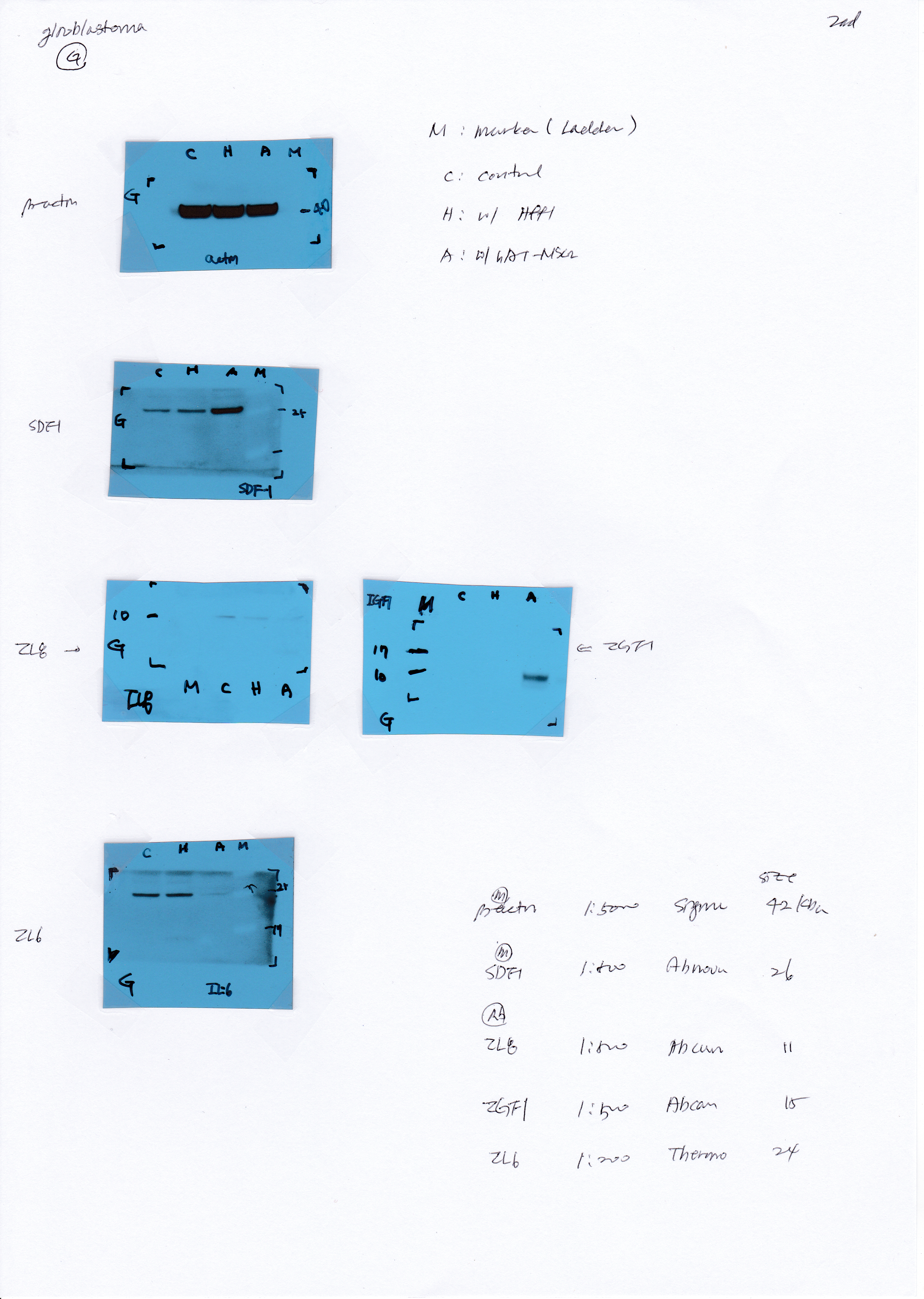

Supplement: S1 File — Cyto-chemokine ligand protein expression in brain tumor-initiating cells (BTICs) after co-culture with human adipose tissue-derived mesenchymal stem cells (hAT-MSCs) or HFF1 cells. β-actin was used as protein loading control. The edges of membrane and size (kDa) were marked in blot. The proteins were loaded the marker (M), only BTICs (Control: C), BTICs co-cultured with HFF1 (H), BTICs co-cultured with hAT-MSCs (A) in the order named. In medulloblastoma-BTICs and atypical teratoid/rhabdoid tumors (AT/RT)-BTICs, the blots were arranged in β-actin, SDF-1, IL-8, IL-6 and RANTES. In glioblastoma-BTICs, the blots were arranged in β-actin, SDF-1, IL-8, IGF-1 and IL-6. The patterns of protein expression were the increased expression of SDF-1 and decreased expression of IL-8 in all BTICs co-cultured with hAT-MSCs. In medulloblastoma-BTICs and AT/RT-BTICs, the expression of RANTES is increased, but that of IL-8 is not changed. In glioblastoma-BTICs co-cultured with hAT-MSCs, the expression of IGF-1 is higher but that of IL-6 is lower. (DOCX) [file pone.0132877.s001.docx]
